# Applying and Extending the FITT Framework to Identify the Challenges and Opportunities of Successful eHealth Services for Patient Self-Management: Qualitative Interview Study

**DOI:** 10.2196/17696

**Published:** 2020-08-12

**Authors:** Sari Kujala, Elske Ammenwerth, Heta Kolanen, Minna Ervast

**Affiliations:** 1 Department of Computer Science Aalto University Espoo Finland; 2 UMIT – Private University for Health Sciences, Medical Informatics and Technology Hall in Tirol Austria; 3 HUS IT Management HUS Helsinki University Hospital Helsinki Finland; 4 Development Unit Hospital District of South-West Finland Turku Finland

**Keywords:** interview, implementation, adoption, patient self-management, organization

## Abstract

**Background:**

The number of public eHealth services that support patient self-management is rapidly increasing. However, the implementation of these eHealth services for self-management has encountered challenges.

**Objective:**

The purpose of this paper was to analyze the challenges and opportunities of implementing eHealth services for self-management by focusing on the fit between the technical solution and clinical use.

**Methods:**

We performed in-depth interviews with 10 clinical project coordinators and managers who were responsible for developing and implementing various eHealth services for self-management interventions in five university hospitals in Finland. The results were analyzed using content analysis and open coding. The Fit between Individuals, Task, and Technology (FITT) framework was used to interpret the findings.

**Results:**

The implementation of self-management services involved many challenges related to technical problems, health professional acceptance, patient motivation, and health organization and management. The implementers identified practices to manage the identified challenges, including improving the design of the technology, supporting health professionals in the adoption of the eHealth services, changing the work processes and tasks, involving patients, and collectively planning the implementation inside an organization. The findings could be mostly attributed to the dimensions of the FITT framework.

**Conclusions:**

The FITT framework helped to analyze the challenges related to the implementation, and most of them were related to poor fit. The importance of patients as stakeholders in eHealth services for patient self-management needs to be highlighted. Thus, we propose that patients should be added as a different type of individual dimension to the FITT framework. In addition, the framework could be extended to include organization and management in a new context dimension.

## Introduction

### eHealth for Self-Management

In many countries, the number of public eHealth services that support patient self-management is rapidly increasing. Barlow et al [1] define self-management as an individual’s ability to manage the symptoms, treatment, physical and psychosocial consequences, and life changes inherent in living with a chronic condition. According to their review, self-management interventions benefit participants’ well-being. Diabetes and heart failure interventions seem to be particularly effective [2].

However, many studies also report that the implementation of eHealth services that support self-management has encountered challenges, such as motivating patients and health professionals [3-6]. The implementation challenges have also led to limited adoption by patients and health professionals and their use of the services [3,4,7].

Health care professionals have many concerns related to self-management services and their professional roles in these new situations [8,9]. They especially worry about whether patients are willing and able to use these new services. Thus, they may not be willing to introduce new eHealth services to patients, but their endorsement increases patients’ trust in a technical solution [10] and greatly impacts patients’ initial adoption and continued use of eHealth services [11].

Several literature reviews have identified factors that facilitate or hinder the successful implementation of eHealth services [12-14]. While these studies have identified many good implementation practices, such as the importance of leadership support, the suitability of the practices and approaches depends on the context [12,15]. The self-management context has rarely been studied. Thus, this study sought to gain deeper knowledge in this area through a qualitative interview study.

### Fit Between Individuals, Task, and Technology Model

Several implementation models can be used to analyze barriers and facilitators that occur during implementation [16]. We applied the *Fit between Individuals, Task, and Technology* (FITT) framework developed by Ammenwerth et al [17], as it helps analyze the factors that influence the success or failure of information technology (IT) implementation in a health care setting. The FITT framework has already been shown to be useful in several case studies [18-21].

The FITT framework is based on the idea that IT adoption in a clinical environment depends on the optimal fit or interaction between the attributes of three fit dimensions: the individual users, the technology, and the clinical tasks and processes [17]. An individual represents a single user or a user group. Technology includes the interaction of the various tools needed to accomplish a given task. The task comprises all working processes and tasks that need to be completed. Organizational aspects are included either as part of the individual dimension or part of the task dimension. One of the FITT framework’s specific strengths is its focus on the interaction between the user and the task [20], as issues related to IT support of professionals’ workflows are the most frequently reported failure factors of eHealth interventions [22].

Compared to other frameworks, such as the Consolidated Framework for Implementation Research (CFIR) [23], the FITT framework differs in its addition of the interaction aspect. While the CFIR suggests that the characteristics of individuals and interventions influence implementation, the FITT framework considers the interactions between the characteristics of individuals and technical interventions. According to the FITT framework, the influence of technological interventions also depends on the individuals’ motivation, knowledge, and training.

Tsiknakis and Kouroubali [18] used the FITT framework in their case study and reported that the model provided a structured way to explain the reasons for the success or failure of IT systems and eHealth services. Prgomet et al [19] also found that the health professionals’ use of technology was related to the fit between users, tasks, and technology. However, they identified additional environmental factors, such as the temporal rhythms of a ward or space limitation, and proposed that the FITT framework should be extended to include an environment dimension as well. In addition, the FITT framework has been successfully used to analyze different stakeholders’ perceptions of eHealth, such as those of nurses [24], case managers [20], and patients [25].

In summary, the FITT framework has been useful in analyzing implementation barriers and facilitators in different case studies. However, self-management eHealth services are novel for patients and health care professionals and require complex changes in clinical care [6]. Both patients and health care professionals need to be motivated to use these services, and there remains a limited understanding of how these services should be implemented.

This paper applies the FITT framework to analyze the implementation experiences of 10 clinical project coordinators and managers who were responsible for implementing digital care paths supporting patient self-management in five university hospitals. In a study by Murray et al [26], staff charged with implementing eHealth initiatives had a deep understanding of the barriers to and facilitators of success. Thus, we specifically collected information from the clinical project coordinators and managers who were responsible for developing and implementing various eHealth services for self-management. As the implementation of eHealth services was in the early phase and health care professionals had a key role in endorsing and engaging patients [9,10], project coordinators focused more on introducing the services to health care professionals than to patients.

### Study Aims

The aims of this study were (1) to identify the specific challenges to implementing eHealth services for self-management and opportunities to manage these challenges and (2) to evaluate how well the FITT framework explains the identified challenges in the context of self-management eHealth services. The findings provide a better understanding of the factors that influence the implementation of eHealth services for self-management interventions and extend the FITT framework to explain the success of eHealth service adoption.

## Methods

### Study Setting

In Finland, the objective of the national eHealth and eSocial Strategy 2020 is to support the active role of citizens in promoting their own well-being, preventing health problems, self-assessing the need for services, and independent coping [27]. As a part of the strategy, an eHealth portal, HealthVillage.fi, was developed by the joint Virtual Hospital 2.0 project among five Finnish university hospitals. The project was funded by the hospitals and the Ministry of Social Affairs and Health. The Virtual Hospital 2.0 project was funded from 2016 to 2019 and was coordinated by the HUS Helsinki University Hospital (referred to simply as HUS). The goal was to raise the quality of specialist health care services and improve their accessibility with the use of digital technology.

The coordinating HUS developed the technical platform for developing eHealth services for citizens, patients, and professionals. The joint project provided guidelines for planning the content and implementation. Using the guidelines, the services were developed by a multi-professional team usually including physicians and nurses.

The eHealth portal was developed in two phases. First, an open-access eHealth portal was developed to offer information, advice, self-care instructions, and symptom navigators for Finnish citizens and patients. The portal includes over 20 eHealth services, called hubs, that focus on specific patient or disease groups, such as neurological diseases.

Second, hospital-specific digital care paths only open to invited patients of a care unit were developed [28], and the first two paths were opened in November 2017. The digital care paths were designed to supplement and offer alternatives to the traditional treatment paths. The functionalities depended on the patient group, but they included patient instructions, exercises, self-monitoring and symptom assessment, and secure messaging. A digital path could be for short-term treatment, such as surgery, or for longer-term care of a chronic disease. The team planned the functionalities and developed the content. The project coordinators were trained for their positions, and they were responsible for adding the content to the platform and implementing the new service. Each project coordinator was supported by a developer partner from an IT organization.

Table 1 summarizes the digital care paths and their functions for which the participants were responsible for implementing in their organizations. Three of the digital care paths were to support patients during preparation for a surgery—mitral valve surgery, cervical spine surgery, and pacemaker surgery—and during postsurgical care. Three were short-term digital care paths for couples receiving in vitro fertilization treatment, pregnant women, and women with gestational diabetes. Two were long-term digital care paths for patients with spinal cord disability and rheumatism.

According to the project coordinators, the main goals of the new digital care paths were to improve the quality of service provided to patients and to support self-management and communication between patients and care personnel. Patients were expected to be better informed and require less guidance when using the paths. Nurses could also receive information from patients, monitor them, and perform preventive interventions when needed. Organizations also aimed to minimize costs by reducing the number of phone calls and moving the communication to digital messaging.

As the digital care paths had been used from 1 to 10 months, the technical platform was still under development, the number of patients using a service remained low, and all services were in the early stage of implementation. The development of the technical platform was continuously developed based on professionals’ and patients’ feedback using an agile approach.

**Table 1:** Description of digital care paths.

Digital care path	Functions	Starting date (first patient entered)	Estimated potential number of patients	Real number of patients between the starting date and 11/3/2019 (path ended or still ongoing)
Mitral valve surgery	Information Health questionnaires	4/2018	150 per year	177
Cervical spine surgery	Messaging Self-management information Appointments Anamneses forms	10/2018 piloting	500 per year	244
Pacemaker surgery	Information before pacemaker implantation and answers to the most frequently asked questions Messaging	12/2018	300 per year	214
In vitro fertilization	Appointments and questionnaires Information and instructions Messaging	11/2017	400 per year	597
Pregnancy	Appointments Information on practicalities and fetal screening	11/2017	10,000 per year	17,229
Gestational diabetes	Messaging, self-management information, diary, tasks, and tests Mobile app	1/2019	200 per year	239
Spinal cord disability	Messaging and sending pictures Anamneses forms Ability-to-function forms Symptom diaries Remote consultations	10/2018 piloting	200 per year	200
Rheumatology	Information about the clinic and care and answers to the most frequently asked questions Messaging	1/2019	Thousands per year	1951

### Study Participants

The participants were selected by purposive sampling. The goal was to cover a variety of experiences of different eHealth services and contexts from a project management point of view. The inclusion criterion was a responsibility to implement a digital care path in a care unit. Implementers were chosen for this study, as they have experience planning and managing implementation, and they observe factors that influence implementation [26]. The participants were recruited by a development manager of HUS who had contact with project coordinators and managers.

A total of 10 participants were interviewed from five hospitals (see Table 2). In total, 7 of them were nurse project coordinators who were responsible for the practical implementation of the digital care paths in their unit. In addition, 1 was a physician project manager who was leading an implementation project alongside her clinical work. As one of the hospitals had decided to postpone the development and implementation of the digital care paths, their development manager and 1 technical manager were also selected to be interviewed to reveal their experiences.

**Table 2:** Details about the study participants.

No.	Role	Expertise	Responsibility	Hospital ID
1	Development manager	Economics, change management, and implementation	Managing digitalization of health services	1
2	Technical product owner and project coordinator	Software engineering	Project manager of digital care paths	1
3	Physician project manager	Medicine	Planning and implementing a digital care path	2
4	Nurse project coordinator	Nursing	Planning and implementing a digital care path	2
5	Nurse project coordinator	Nursing	Planning and implementing a digital care path	2
6	Nurse project coordinator	Nursing	Planning and implementing a digital care path	2
7	Nurse project coordinator	Nursing	Planning and implementing a digital care path	2
8	Nurse project coordinator	Nursing	Planning and implementing a digital care path	3
9	Nurse project coordinator	Nursing	Planning and implementing a digital care path	4
10	Nurse project coordinator	Nursing	Planning and implementing a digital care path	5

All the participants were women, and their age ranged from 33 to 53 years. None of the project coordinators or the project manager had previous experience developing or implementing eHealth services.

### Data Collection and Analysis

One interviewer completed semistructured interviews with each participant. In total, 2 participants wanted to have a pair interview. The interviewer met the participants in their office or performed the interviews through videoconferencing. The interview included questions from two main themes:

1. The challenges of the implementation.
2. Opportunities to manage these implementation challenges.

In addition, background information about the interviewee; information about the digital care path, planning, and implementation; and patient feedback were collected.

The interviews were conducted by the first author (SK) from May 2018 to November 2019. The interviews lasted from 1 to 2 hours, and they were audio recorded and transcribed for analysis. In addition to interview data, a documented patient feedback survey report was used as an information source.

After each interview, the main observations were recorded as notes. The analysis was performed in two stages. In stage 1, open coding was used to identify themes in the data. Using in vivo coding, the respondents’ words were used to define the themes to ensure that the themes represented the original meaning of the respondents. One of the authors created a coding scheme using a subset of four interviews. The coding scheme was checked by two other authors before it was used to code the rest of the interviews. Any new themes that emerged in subsequent coding were added to the coding scheme. Finally, the number of interviewees mentioning a theme was calculated. As the development manager and technical manager were interviewed together and shared experiences in the same hospital that postponed the implementation of digital care paths, the analysis of their responses was combined.

In stage 2, the FITT framework [17] was used as a deductive coding framework [29] to place the identified themes in a theory context. The themes identified in stage 2 were categorized into the FITT framework dimensions of task, technology, and individuals, as shown in Tables 3 and 4. The attributes of the dimensions identified by Ammenwerth et al [17] were used to support this categorization.

**Table 3:** Challenges of implementing eHealth services for self-management.

Dimension and themes		Mentions (n=9), n (%)
**Individual-technology fit: health professionals**		
	Problems with usability, technical problems, and missing functionalities	8 (89)
	Resistance, lack of use, and difficulty changing professionals’ work processes	7 (78)
	Professionals not informing patients about the eHealth services	3 (33)
	Negative previous experiences with information systems	1 (11)
	Lack of training	1 (11)
	Lack of technical support	1 (11)
**Individual-technology fit: patients**		
	Problems with usability and missing functionalities	7 (78)
	Lack of use	4 (44)
	Lack of active patient participation in planning	1 (11)
**Health professional–task fit**		
	Extra work caused by insufficient interoperability	3 (33)
	Bad fit with the work processes	2 (22)
**Organization and management–technology fit**		
	Lack of knowledge about the possible technical functionalities	3 (33)
	Lack of resources	3 (33)
	Lack of management support	2 (22)
	Unclear roles during implementation	1 (11)
	Failure of the initial technical platform to fit the organization’s goals and processes	1 (11)

**Table 4:** Practices for managing the challenges of implementation.

Dimension and practices		Mentions (n=9), n (%)
**Individual-technology fit: health professionals**		
	Involving all the stakeholders, professional groups, and a technical expert in planning the service	2 (22)
	Testing and piloting the eHealth services before implementation	4 (44)
	Repeatedly informing health professionals about the implementation, changes, and the influence of the new services to their work for an extended period via unit meetings, training, personal contacts, and laminated instructions	4 (44)
	Proactively responding to health professionals’ concerns	1 (11)
	Involving frontline leaders and health professionals in planning the services and implementation is needed to create buy-in	3 (33)
	Providing adequate introductory knowledge, repeated training, and personal guidance as well as a test environment, which is required to train professionals	4 (44)
	Training a superuser to encourage health professionals and support implementation	1 (11)
	Proceeding slowly and gradually, so professionals have time to adjust to and practice using the new services	2 (22)
	Reserving extra personnel resources and time for the changing tasks	1 (11)
	Providing technical support with a responsible person when needed	1 (11)
**Individual-technology fit: patients**		
	Identifying a patient group that can benefit from an eHealth service and having a patient point of view	2 (22)
	Involving patients early on and creating new methods needed to motivate patient participation	2 (22)
	Informing patients in an interesting way and providing leaflets or other marketing materials	2 (22)
	Collecting constant feedback from patients and improving the service through the use of questionnaires, contacting patients for further details to create a partnership, and request for feedback from patients that did not use the service	3 (33)
	Encouraging health professionals to discuss the digital service when meeting a patient and to reserve digital appointments with the patient	2 (22)
	Offering technical support during problems	1 (11)
**Health professional–task fit**		
	Identifying the current work processes and needs	1 (11)
	Fitting the eHealth service plans to current care processes to support and ease health professionals’ work, reduce the number of phone calls, increase remote work, and increase interoperability of the systems so that health professionals can view patient information from one system and do not need to record the same information twice	5 (56)
	Ensuring the service is quick, easy, and effortless to request and use	5 (56)
	Planning changes in the work processes well in advance and piloting the services to test the new processes and show the benefits of the service	1 (11)
**Organization and management–technology fit**		
	Identifying the needs early	1 (11)
	Fitting the eHealth service plans to the technical possibilities, including demonstrations and examples of existing services to help illustrate the possibilities	2 (22)
	Evaluating the work cost of implementation, a responsible person, resources needed, and the potential benefits	2 (22)
	Planning the implementation carefully in advance and defining the roles and responsibilities of the participants, changes, ways of relieving resistance, and solutions to problems	5 (56)
	Involving an active multi-professional team	1 (11)
	Indicating more than one person as a spokesperson to support the implementation, especially frontline leaders, who were considered important in influencing their subordinates’ commitment and providing resources for implementation	2 (22)
	Involving and informing top management to provide support and resources and ensure the availability of spaces and devices	2 (22)
	Identifying measures of impacts and making baseline measurements in the very beginning	1 (11)

### Ethics

The interviewees received oral and written information about the study and its voluntary nature before the interviews. Written informed consent was obtained from all participants. The study protocol was reviewed and approved by the Ethical Review Board of Aalto University, Finland.

## Results

### Challenges of Implementing eHealth Services for Self-Management

Table 3 summarizes the challenges that the participants experienced in implementing the eHealth services. We categorized the challenges according to the FITT model dimensions. Most of the challenges were related to poor fit between individuals and technology, such as health professionals suffering usability problems, technical problems, and missing functionalities. Health professionals were reported to be critical of new services because they had had negative experiences with information systems. One of the first digital paths had a challenging start, as nurses needed to solve the usability problems that patients faced. Nurses had no training on how to use the service, and technical support was not yet available.

Many of the health professionals were not willing to use the new eHealth services. One of the participants described, “The resistance over changing practices surprised me most and how long it has been continued.” She also said that not all the nurses understood how this service was going to help with their work; hence, their motivation to use it was low. Only 3 of the participants did not mention any professionals’ resistance or lack of use, but their eHealth services were only used by the developing team, which consisted of 2-10 health professionals. Thus, it seemed to be more challenging to introduce a service to larger user groups. In total, 3 participants mentioned that the group of health professionals who had not participated in the development team demonstrated the most resistance.

One interviewee described that it was very challenging to reach and inform all the health care professionals as they worked in three shifts. Physicians forgot to use the digital path as a tool and tell their patients about the new service. In addition, another participant said that physicians used the old paper-based approach and were reluctant to use the digital path, which appeared to be slower. Nurses also forgot to reserve digital appointments for the patients.

The services included a patient feedback survey, and all except one implementation project received feedback from patients through the survey. The feedback provided was positive, and the services were evaluated to be useful. For example, the information received was seen to support preparation for an operation. Patients gave negative feedback related to difficult registration, problems in use, slow or confusing services, and missing functionalities.

In the documented survey, 15 out of 17 patients (88%) rated that they were *satisfied* or *rather satisfied* with the service, and all of them agreed that the service was useful. However, they reported missing instructions, impractical registration, slowness, and other difficulties that made using the service cumbersome or disrupted the service.

The number of patient users was also low in some cases. Health professionals often did not actively use the new eHealth services, and they were also passive in introducing the services to patients and motivating them to use the services. Patients did not provide much feedback, and the number of patients who completed the questionnaires was low. One of the interviewees said that they were not able to find patients who were willing to participate in the design workshops. Thus, in the context of online self-management intervention, patients need to be engaged in both using and designing the services. We, therefore, divided the individual dimension to separately cover health professionals and patients.

Some participants identified fit problems between technology and tasks. Mostly, the lack of interoperability created extra work for health professionals, but in 2 cases, participants identified that the process required by the eHealth service did not fit the work processes.

In addition to the FITT model’s dimensions, there were challenges related to poor fit between the organization and management and the technology. For example, it was unclear how the technical platform could be used in the organization and what benefits exist. In some cases, there was insufficient management support or available resources for implementation. For example, one of the interviewees found it challenging to support others in the implementation, as she did not have reserved work hours for it. She felt that management should also have provided resources for implementation and use and not only for the development of the digital path.

The development of the technical platform was in one hospital at the beginning; the initial version of the platform did not fit the organization’s goals and processes. The usability and interoperability of the customer management system was considered to be poor. Consequently, the implementation of any new eHealth services using the technical platform in this hospital was halted until the platform was further developed.

### Opportunities to Manage the Challenges of Implementing eHealth Services for Self-Management

Table 4 shows the practices that the participants found successful or recommended for managing the identified challenges and supporting the implementation of eHealth services. Many of the practices were related to improving the design of the eHealth services by involving all the stakeholders, testing and piloting the service, and collecting feedback. Management practices, such as informing health professionals, responding to their concerns, and supporting them in the change, reduced health professional resistance. In addition, involving and informing patients and collecting their feedback was considered important.

Participants found that task-technology fit could be improved by aligning the new service to the current work processes and needs and designing a service that is usable and effortless to use. To improve the fit between the organization and the technology, participants saw a need to identify a relevant problem that the technology was able to solve and the benefits that could be produced and to evaluate the costs related to implementation. Many participants also learned that implementation needed to be well planned and organized collectively inside the organization.

## Discussion

### Principal Findings

The study shows three major findings:

1. The implementation of self-management services involved many challenges related to technical problems, health professional acceptance, patient motivation, and health organization and management.
2. The implementers had identified practices to manage the identified challenges by improving the design of the technology, supporting health professionals in the technology adoption, changing the work processes and tasks, involving patients, and planning the implementation collectively inside the organization.
3. The challenges and practices could be mostly attributed to the dimensions of the FITT framework. However, the findings suggest that patients should be added as a different type of individual dimension, and organizations and management should be added as new dimensions to the FITT framework.

### Challenges of Implementing eHealth Services for Self-Management

In line with an earlier case study [6], it was challenging to introduce self-management services to health care professionals, change their work practices, and motivate patients to use the service. The poor fit of tasks, the technology, and individuals troubled health care professionals. As shown by previous studies, these issues are remarkably common barriers to eHealth interventions [22].

Furthermore, the technology needs also fit the organization, and management has an important role, as identified in previous studies [12,30]. The technical solution fit the strategy and operation model of the organization, and successful commitment requires commitment and resources from management. Many of the challenges were interrelated. For example, if the new service did not fit the work processes of the health professionals, they did not inform patients about the service, which resulted in a lack of use among both professionals and patients.

### Opportunities to Manage the Challenges of Implementing eHealth Services for Self-Management

Most of the identified opportunities to handle the challenges were related to improving the fit between tasks, the technology, and individuals. Better fit can be achieved by designing better technical solutions and usability to support patients, health professionals, and tasks. Therefore, it is important to foster stakeholder involvement and user-centered design in future projects [31-33]. Current work processes and the needs of users should be understood at the beginning of the design. Different stakeholders also need to be involved to identify their needs and encourage buy-in. In the studied cases, health professionals served as designers and implementers. Thus, health professionals were involved, but this situation also created new challenges, as the professionals had no experience in user-centered design or implementations.

In addition, many of the opportunities to handle the identified challenges were related to the management and the organization. As shown by previous studies, the way eHealth services are introduced is also important for implementation success [8,12]. Top management support and a shared strategic vision of the new technology are often reported to support implementation [30], but the results from this study also pointed out the need for the collective support of frontline leaders and other spokespersons.

### Extension of the FITT Framework to Better Cover the Patient as an Individual and Organizational Context

**Figure 1:**
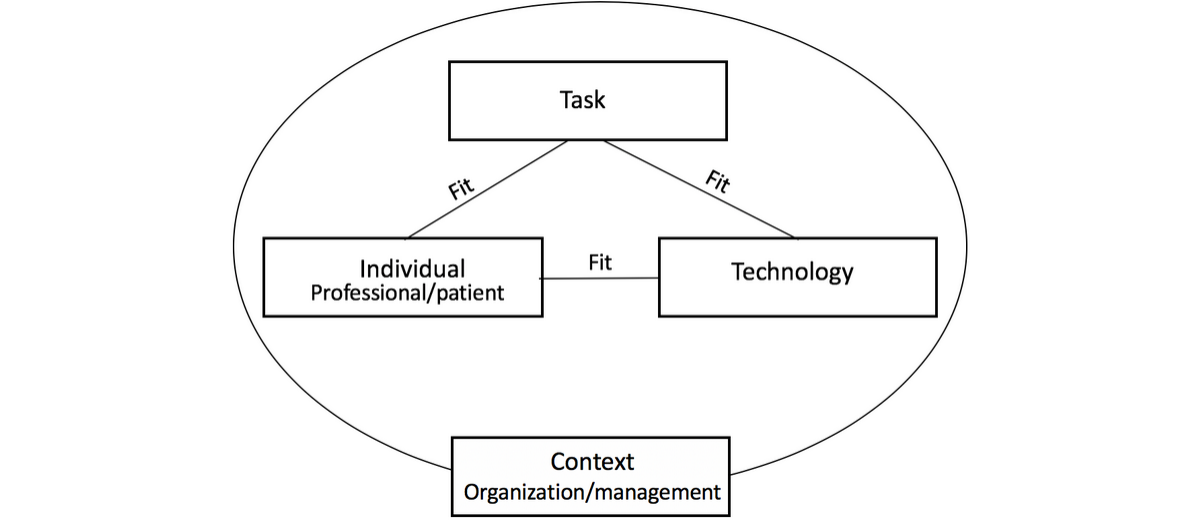
Extended FITT (Fit between Individuals, Task, and Technology) framework.

Overall, the findings were well interpreted with the dimensions of the FITT framework (see Figure 1). However, the role of patient stakeholders in self-management needs to be highlighted, and we propose that both health professionals and patients are represented in the individual dimension.

In the original FITT framework, the organization is mentioned as one of the attributes of the individual level. However, previous research shows that in complex implementation projects, there are also higher-level contextual factors, such as the health care system, the social climate, and economic and political issues, that are important to consider [34,35]. Additionally, many of the identified challenges and opportunities in this study were not related to individuals but to organizations and management in a broader context. For example, if management did not provide resources for implementation, training, and planning, individuals were not supported to change their work processes. Furthermore, our results imply that it is challenging for an individual to manage implementation. Instead, the implementation work and introduction of a service to patients need to be supported collectively.

Thus, we propose that organizations and management be represented by a separate context dimension, as shown in Figure 1. Unlike the findings of Prgomet et al [19], our findings did not demonstrate environmental issues, which is probably because we did not observe professionals’ behaviors in detail. However, we suggest that the environment is a part of the new context dimension.

The extensions we proposed to the FITT model are not specific to self-management services. However, these kinds of services influence health professionals’ and patients’ interactions in a novel way; thus, the extensions especially support the analysis of the organizational efforts to engage patients in care.

The combination of the FITT framework and the Clinical Adoption Meta-Model [36] may help to further explain the progress of the implementation from one phase to the next over time. In this study, one organization did not proceed to the initial phase, where the service was available to professionals and patients, because the technology fit poorly with the organization’s strategy and operational model, as interoperability and information safety were not considered acceptable. Other organizations made the new services available, but there were challenges in initiating use of the services due to usability and interoperability reasons. One of the services was also challenging to use, as it did not fit the work processes and needed to be redesigned. Without sufficient use, there were no clinical or health behavior changes or positive outcomes.

### Comparison With Prior Work

Our results highlight the complex relationship between different factors and stakeholders that influence the success of implementation. The larger the number of users, the more complicated the implementation was in this study. In a literature review, Ludwick and Doucette [37] suggested that large organizations should use an incremental approach due to the complexities associated with their size. In addition to complexity, the larger number of users made participation in planning the new services and the related work processes more difficult. As Granja et al [22] identified in their literature review, this study observed that it was especially challenging to change health professionals’ work processes. Our results suggest that the participation of health professionals in the change of their work processes could support the change process.

In the case of eHealth services for self-management, patients are important stakeholders. Urowitz et al [3] found that patients were not always active in an online diabetes self-management portal, and their use declined over time. Thus, patients should be involved early to ensure the quality of the eHealth services and should be supported during adoption of the services. Health professionals play an important role in endorsing the new services to patients [10].

Our results imply that although many of the challenges of implementation are similar to those of different eHealth innovations [14,38], new approaches are clearly needed to handle the challenges and lack of fit in practice. In the self-management context, the fit between individuals and the technology needs to be improved both from health care professionals’ and patients’ points of view. Both groups need to be involved in the design, and they should be well informed. Their feedback should also be constantly collected. In improving the fit between the health care professionals and the tasks, the goal should be to improve the current work processes and ease health care professionals’ work.

### Limitations

A limitation of this study is that we only studied the perspectives of project coordinators and managers who were responsible for implementing the new online self-management services. As our study relied on the implementers’ reporting of health care professionals’ reactions, the health care professionals’ characteristics influencing the implementation were not identified, as they were identified in an interview study by Ciere et al [6]. To gain a more comprehensive understanding of the challenges and practices, other stakeholders, such as patients and health care professionals, should be studied in the future as well.

The number of interviewees was relatively low. However, the goal was to gain qualitative insight into the challenges and opportunities in the implementation projects, and data saturation was reached, meaning that not much new information (eg, a single new theme) could be identified in an additional interview [39]. The participants were also relatively inexperienced in implementing eHealth services, and the hospitals were all in Finland. Implementers working in other organizations may face different implementation challenges, and the generalizability of this study is restricted to this specific context.

The level of health care professionals’ resistance can vary during implementation [40-42]. Our study provided only a cross-section of the implementation process, and a better understanding of the timing of the challenges and best implementation practices are needed. Further studies should clarify how health care professionals and especially patients can be better supported in the adoption of eHealth services.

## Abbreviations

CFIR: Consolidated Framework for Implementation Research
FITT: Fit between Individuals, Task, and Technology
HUS: HUS Helsinki University Hospital
IT: information technology
